# In Vitro Digestion Patterns of Advanced Glycation End Products and α-Dicarbonyls in Biscuits and the Modulatory Effects of Ferulic Acid and Epicatechin

**DOI:** 10.3390/foods14081429

**Published:** 2025-04-21

**Authors:** Xiaoxiang Peng, Huiyu Hu, Yuwei Liu, Jia Li, Yilun Huang, Haiwa Wang, Ziyi Wang, Yuting Wang

**Affiliations:** State Key Laboratory of Food Science and Resources, China-Canada Joint Lab of Food Science and Technology (Nanchang), Nanchang University, 235 Nanjing East Road, Nanchang 330047, China; 15708918997@163.com (X.P.); ncuhhy@ncu.edu.cn (H.H.); 417900240031@ncu.edu.cn (Y.L.); jlee0423@163.com (J.L.); hyl675322189@sina.com (Y.H.); ncuspywanghaiwa@163.com (H.W.); 18770819180@163.com (Z.W.)

**Keywords:** in vitro gastrointestinal digestion, polyphenols, process contaminants, adducts

## Abstract

The dietary intake amount of processing contaminants does not reflect their actual exposure risk due to interactions with the food matrix during gastrointestinal processes, which significantly modulate their bioaccessibility. This study systematically investigated the in vitro digestion patterns of advanced glycation end products (AGEs) and α-dicarbonyl compounds (α-DCs) in biscuits and the modulatory effects of ferulic acid and epicatechin. The results demonstrated that more than 80% of AGEs and α-DCs were present in the bioaccessible fraction of the samples after intestinal digestion. Ferulic acid (FA, 0.05%, *w*/*w*) significantly increased the AGEs content in the bioaccessible fraction after intestinal digestion compared to control samples. Conversely, FA at 0.2% and 0.5%, as well as epicatechin (EC) at 0.05%, significantly reduced the glyoxal and 3-deoxyglucosone levels during oral digestion and significantly increased these contaminants contents after gastric digestion. The higher the concentration of EC, the lower the level of methylglyoxal during oral and gastric digestion. In addition, we identified the adducts of FA with lysine and the adducts of EC with Nε-Carboxymethyl-lysine using LC-QTOF-MS, demonstrating the reactivity between polyphenols, amino acids and contaminants. This study provides guidance and suggestions for mitigating dietary exposure to AGEs and α-DCs.

## 1. Introduction

Cereals are rich in carbohydrates, which are the main source of dietary energy [1]. During hot processing, carbohydrate-rich foods form various thermal processing contaminants, such as acrylamide (AA), α-dicarbonyl compounds (α-DCs), and advanced glycation end products (AGEs), through *Maillard* and caramelization reactions.

Among them, dicarbonyl compounds, a key intermediate in the *Maillard* reaction, and their derived AGEs may be associated with genotoxicity, neurotoxicity and chronic diseases, such as diabetes, and may pose a health hazard and lead to an overall reduction in the nutritional value of food when ingested in the daily diet [2,3,4]. AGEs are complex and diverse, of which Nε-carboxymethyl-lysine (CML) and Nε-Carboxyethyl-lysine (CEL) are well characterized and are typical markers reflecting the level of AGEs in food [5,6]. Highly reactive α-DCs are usually produced in carbohydrate- and protein-rich foods, with glyoxal (GO), methylglyoxal (MGO) and 3-deoxyglucosone (3-DG) being the most studied [3]. Cereals, such as commercial biscuits, have highly variable levels of GO, MGO, and 3-DG, depending on their food ingredients and processing methods [7].

However, the total amount of contaminants ingested does not always reflect the amount of contaminants to which the human body is exposed, and this process can be modulated by the compositional properties of the food matrix and the physiological conditions of the gastrointestinal tract [8]. Formation and elimination of contaminants occur not only during thermal processing but also in the human body. The effect of digestion on contaminants is controversial in the literature. It has been noted that the formation of AGEs is temperature and pH dependent [9], and that the pH of the stomach differs from that of the intestine, which may exert an influence on the levels of contaminants. Thermal processing concomitants that enter the digestive tract may react with other food components in the digestive tract, such as amino acids, proteins, and polyphenols, to form unknown compounds. Hamzalıoğlu and Gökmen discovered that the concentrations of MGO and 3-DG in commercial biscuits decreased during digestion, attributed to the interaction of active DCs with amino acids during digestion, and identified adducts of amino acids and DCs [10]. Li et al. detected the formation of adducts between reactive carbonyls and polyphenols under simulated physiological conditions [11]. Many studies have demonstrated the capacity of polyphenols to impact the generation of concomitants in thermally processed foods. However, the mechanism by which they affect concomitants after entry into the gastrointestinal tract is not yet fully understood; it is important for human health to further investigate the alterations and mechanisms that occur in the gastrointestinal tract of thermal processing contaminants.

The presence of AGEs and α-DCs in both bioaccessible and non-bioaccessible fractions was monitored by means of a standardized in vitro digestion protocol INFOGEST (which primarily simulates the oral cavity, stomach, and duodenum) [12]. Experiments explored the changing patterns of AGEs and α-DCs in the bioaccessible and non-bioaccessible fractions of biscuits (a typical representative of cereal grains) after consumption, and discussed the effects of different concentrations of ferulic acid and epicatechin on thermally processing contaminants during digestion and the identification of adducts by high-resolution mass spectrometry to explore the mechanisms by which polyphenols affect contaminants. This study provides a theoretical basis for polyphenols in the gastrointestinal tract affecting the contaminants formed during the thermal processing of food, and it can also provide guidance for better assessing the bioaccssibility of contaminants.

## 2. Materials and Methods

### 2.1. Chemical and Materials

Breakfast biscuits (original flavor, Kasly Food Group Co., Ltd.) were purchased from the local market in Nanchang. FA (analytical standard), gall (≥45% purity), D-(+)-Glucose (≥99.5%, GC), L-Lysine (analytical standard), glyoxal solution (40 wt% in H_2_O, 8.8 M), methylglyoxal (40 wt% in H_2_O), and α-amylase (from bacterial) were acquired from Macklin Biochemical Co., Ltd. (Shanghai, China). EC (≥98%) was obtained from Shanxi hafo Bio-Technology Co., Ltd. (Xian, Shanxi, China). CML, CEL, d_4_-CML, d_4_-CEL, GO and MGO were purchased from Toronto Research Chemicals (North York, ON, Canada). 3-DG (≥75%), quinoxaline (≥99%), 2-methylquinoxaline (≥97%), 5-methylquinoxaline (≥97%), o-phenylenediamine (OPD, ≥98%), porcine pepsin (≥2500 U/mg), and pancreatin (from porcine pancreas, 8 × USP specifications, ≥200 units/mg) were acquired from the Sigma-Aldrich Company (St. Louis, MO, USA). Formic acid (eluent additive for UPLC/LC-MS) was acquired from Anpel Experimental Technology Co., Ltd. (Shanghai, China). Deionized water was acquired from the Watson Group Ltd. Chloroform (analytical grade), sodium borohydride (NaBH_4_), and borax (Na_2_B_4_O_7_10H_2_O) were acquired from Sinopharm Chemical Reagent Co., Ltd. (Shanghai, China). All other chemicals, solvents and reagents were of analytical grade. If not otherwise specified, water was used as the solvent when preparing solutions.

### 2.2. In Vitro Digestion of Food Systems

The biscuits performed in triplicate were simulated in vitro gastrointestinal digestion (oral, gastric and duodenal) according to the INFOGEST protocol described by Brodkorb’s scheme, with minor adjustments [12]. Sealed bottles with aluminum caps and glass (Senlan Glass Products Co., Ltd., Xuzhou, Jiangsu, China) were used for digestion and a water bath shaker (Huabang Instrument Manufacturing Co., Ltd., Changzhou, China) was used to incubate samples. The reaction was terminated at all stages by immediately transferring the samples to an ice bath.

To simulate oral digestion, 5 g of food powder was dissolved in 5 mL of distilled water and polyphenols were added as required. The samples were mixed with 4 mL of simulated salivary fluid and 25 μL of 0.3 M CaCl_2_. Then, 0.5 mL of α-amylase enzyme solution (15 mg, 50 units/mg) was added and the pH of the mixing system was adjusted to 7 by using 6 M HCl or 1 M NaOH. The volume was adjusted to 10 mL with water then incubated at 37 °C and 100 r/min for 2 min.

To simulate gastric digestion, 8 mL of simulated gastric fluid was added to 10 mL of the oral digestion mixture and the pH of the mixing system was adjusted to 2.5. Next, 5 μL of 0.3M CaCl_2_ and 0.5 mL of pepsin solution (16 mg) were added and the pH of the mixing system was adjusted to 3. The volume was adjusted to 20 mL with water. The mixture was reacted for 2 h under the same incubation conditions as oral digestion.

Intestinal digestion was simulated by adjusting the pH of gastric digestion products to 7, then adding 8 mL of simulated intestinal fluid, 40 μL of 0.3 M CaCl_2_, 4 mL of pancreatin solution (500 mg), and 6 mL of gall solution (932.4 mg). The volume was finally adjusted to 40 mL with water and incubated for 2 h under the same conditions as described above, and the pH was adjusted to 7 every 30 min.

After digestion, the soluble fraction and the insoluble fraction were carefully separated by centrifuging at 10,733 *RCF* for 10 min at low temperature. The supernatant was transferred to the volumeter bottle for determining the volume and kept in another tube, whereas the insoluble part was lyophilized and stored at 4 °C pending further analysis.

### 2.3. AGEs Determination by LC-ESI-MS/MS

CML and CEL are considered to be the two most prevalent AGEs. The pretreatment of AGEs was modified slightly following a previously established method [13]. Hexane (5 mL) was added to 200 mg freeze-dried insoluble fraction or 2 mL soluble fraction and centrifuged at 5563 *RCF* for 10 min and mixed thoroughly, and the hexane layer was then removed with a disposable pipette. The remaining samples were blown dry with nitrogen and then transferred to a thick-wall pressure-resistant glass tube. The dried residue was added to 1.5 mL of 0.2 M sodium borate buffer (pH 9.2) and 1 mL of 1 M sodium borohydride solution, and reduced at 4 °C for 8 h. Hydrochloric acid (2.5 mL, 12 M) and nitrogen were added to the sample (110 °C, 24 h) for the reduction reaction. The hydrolyzed sample was filtered, and the volume was adjusted to 25 mL with water. A total of 200 ng d_4_-CML, d_4_-CEL and ultrapure water was added to make the volume 1 mL for the solid phase extraction after the 1 mL sample of the 25 mL hydrolyzed sample was blown dry with nitrogen. Solid phase extraction was performed using CNW Poly-Sery MCX SPE (150 mg, 6 mL, ANPEL Laboratory Technologies Inc., Shanghai, China). After the filler was activated and balanced with 3 mL each of methanol and water, the sample was passed through the column. Flushed the column with 1 mL water to remove the impurities adsorbed on it and eluted with 5 mL 5% ammonia solution in methanol (*v*/*v*). The eluent obtained was blown dry with nitrogen, redissolved with 1 mL water, and filtered with 0.22 μm of water-activated polyether sulfone filter membrane for analysis.

Referring to previous research methods, an Agilent 1290-6460 UHPLC system combined with a Triple-Quadrupole Mass Spectrometer (Agilent Technologies Inc., Santa Clara, CA, USA) was used for quantitative analysis of AGEs in biscuit digestion [14]. Hydro-RP 80 Å column (250 mm × 2 mm, 4 µm, Phenomenex, Torrance, CA, USA) was used as the chromatographic column, and the column temperature was 25 °C. The mobile phase was 0.1% formic acid water (100%), the flow rate was 0.15 mL/min, and the sample size was 1 μL. The internal standard method and MRM mode were used. The quantifying transition ions for CEL, d_4_-CEL, CML, and d_4_-CML were *m*/*z* 219 → 130, *m*/*z* 223 → 134, *m*/*z* 205 → 130, and *m*/*z* 209 → 134, respectively. The qualifying transition ions of *m*/*z* 219 → 84 for CEL, *m*/*z* 223 → 88 for d_4_-CEL, *m*/*z* 205 → 84 for CML, and *m*/*z* 209 → 88 for d_4_-CML were also detected. Meanwhile, electrospray ionization (ESI) was applied in positive mode, and the parameters of the ESI source were as follows: capillary voltage 3500 V, drying gas flow rate 10 L/min, nebulizer pressure 25 psi, sheath gas flow rate 9 L/min, and nozzle voltage 400 V.

### 2.4. α-DCs Determination by LC-ESI-MS/MS

The pretreatment of α-DCs was slightly modified with reference to previous research methods [15]. α-DCs need to be derived into stable quinoxaline by o-phenylenediamine before detection. Two mL of chloroform and 1 mL of methanol were added to 200 mg of freeze-dried insoluble fraction or 2 mL of soluble fraction to remove fat and protein, mixed well, and centrifuged at 5563 *RCF* for 10 min, and the supernatant was then taken out using disposable pipettes. Twenty μL of 39 μg/mL 5-methylquinoxaline solution, 400 μL of 10 mg/mL o-phenylenediamine solution, and 380 μL of phosphate buffer (0.1 M, pH 7.0) were added to 200 μL supernatant. Light was avoided for 3 h after swirling for 30 s, then passed through a 0.22 μm water-activated polyether sulfone filter membrane to be measured.

For the detection of α-DCs by UPLC-QQQ-MS/MS, we referred to the previous research methods and modified them slightly. The detection system for α-DCs was the same as for AGEs and was equipped with chromatographic columns Hydro-RP 80 Å column (150 mm × 2 mm, 4 µm, Phenomenex, Torrance, CA, USA). The methanol (A) and 0.1% formic acid water (B) were eluted at 0.25 mL/min flow rate. The column temperature was 30 °C. The ESI source was the positive mode, and the detection mode was the MRM mode. The quantitative and qualitative ions of GO, MGO, 3-DG were *m*/*z* 131 → 77, 58, *m*/*z* 145 → 77, 92, and *m*/*z* 235 → 199, 177, respectively. The procedure of gradient elution was: 0–4 min (40–70% A), 4–6 min (70% A), 6–8 min (70–40% A), and, 8–10 min (40% A). The relevant parameters of the ESI source were as follows: the drying gas temperature was 300 °C, the capillary voltages were 4000 V, the drying gas flow rate was 12 L/min, the nebulizer pressure was 35 psi, and the temperature and rate of sheath gas were 350 °C and 9 L/min.

### 2.5. Establishment of Glucose–Lysine Simulation System

A glucose–lysine–polyphenol model and a lysine-contaminant–polyphenol model were developed to test whether adducts can form between polyphenols, amino acids and contaminants during digestion. The reactants (10 mM of each) were allowed to react in a simulated environment at pH = 3, 37 °C water bath for 2 h or at pH = 7, 37 °C water bath for 4 h. Finally, they were passed through 0.22 µm of water-activated polyether sulfone filter membranes to be measured.

### 2.6. Detection of Adducts Formed During Digestion In Vitro

Determination of adducts was performed using a Nexera X2 liquid chromatography system (Shimadzu Corporation, Kyoto, Japan) in tandem with AB SCIEX Triple TOF^MS^ 5600+ (S AB SCIEX Corporation, Redwood City, CA, USA). The ACQUITY UPLC HSS T3 100A Column (1.8 µm, 2.1 mm × 100 mm) was used for detection and the temperature of the column was 30 °C. ESI was applied in the positive mode. The mobile phase consisted of 0.1% formic acid water (A) and acetonitrile (B), which was eluted from 5% B to 95% B in 16 min at 0.25 mL/min. Data were acquired in the MRM mode with a scan range of *m*/*z* 50 to *m*/*z* 1500 and a collision energy of 35 V for positive ion mode. The declustering potential was 60 V, the curtain gas pressure was 35 psi, and the drying temperature was 550 °C.

### 2.7. Statistical Analysis

Microsoft Excel 2016 was used for basic compilation of the obtained data results, and Origin 2021 was used for bar graphs. Analysis of variance (ANOVA) was performed using SPSS 27.0 with one-way ANOVA and Dunnett T_3_ test for multiple comparisons at *p* = 0.05 level. Significant differences between the two sets of data were determined using the independent samples *t*-test. Experimental data are expressed as mean ± standard deviation (mean ± SD, *n* = 3) of three independent experiments.

## 3. Results and Discussion

### 3.1. Variation of the AGEs Content During the In Vitro Digestion Process

The *Maillard* reaction between reducing sugars and amino groups in biscuit ingredients produces characteristic flavors and colors, while concomitantly producing AGEs. During the *Maillard* reaction, reducing sugars and amino groups react to form Schiff bases, which undergo rearrangement to yield Amadori products. These intermediates further generate highly reactive dicarbonyl compounds, such as GO, MGO, etc. These unstable compounds further react with amino group of lysine to form AGEs, such as CML and CEL [16].

**Figure 1:**
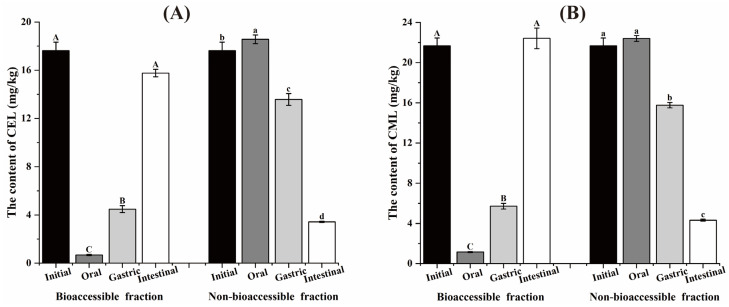
The content of CEL (**A**) and CML (**B**) in the bioaccessible and non-bioaccessible fractions of the sample after oral, gastric and intestinal digestion. Different capital letters and lowercase letters were used to indicate significant differences in the contaminant content of the bioaccessible and the non-bioaccessible fraction after different levels of digestion. The error bars were expressed in SD.

After digestion, the sample exhibited incomplete dissolved. The undissolved residues was regarded as the non-bioaccessible fractions, while water-soluble portion was regarded as the bioaccessible fractions. Figure 1 shows the CEL and CML content in the bioaccessible and non-bioaccessible fractions of the sample after oral, gastric and intestinal digestion without the addition of polyphenols. The initial contents of CEL and CML in the biscuits were 17.6 mg/kg and 21.7 mg/kg, respectively. During digestion, the levels of CEL and CML in the bioaccessible fraction were significantly increased, concomitant with a decrease in the non-bioaccessible. The intestinal digestion led to a significant elevation in total CEL and CML levels, attributed to endogenous formation from AGEs precursors in biscuits. This observation aligns with previous studies, which demonstrated that CML formed by the interaction of fructose with lysine, can be detected following 1 h in vitro intestinal digestion [17]. The presence of AGEs precursors (e.g., reducing sugars and amino acids) in the biscuits formulations likely facilitated AGEs formation during digestion, thereby accounting for the observed increase in total AGEs after intestinal digestion.

**Figure 2:**
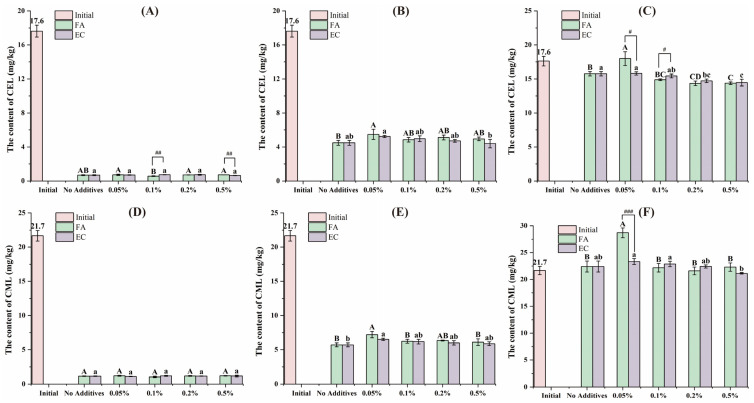
The CEL content of the bioaccessible fraction of the samples after oral (**A**), gastric (**B**) and intestinal (**C**) digestion, and the CML content of the bioaccessible fraction of the samples after oral (**D**), gastric (**E**) and intestinal (**F**) digestion. Different capital letters and lowercase letters were used to indicate significant differences between different concentrations of FA and EC. The # indicates a significant difference between FA and EC at the same level of digestion and concentration (#: *p* < 0.05, ##: *p* < 0.01, ###: *p* < 0.001). The error bars were expressed in SD.

During digestion, a substantial portion of the AGEs in the samples were transferred to the bioaccessible fraction, especially after intestinal digestion, where 80% of the AGEs were detected. In contrast, after oral digestion, AGEs were predominantly existed in the non-bioaccessible fraction. Figure 2 shows the CEL and CML concentrations in the bioaccessible fraction following after oral, gastric and intestinal digestion. For samples intervened with polyphenols, neither FA nor EC significantly affected the concentrations of CEL and CML following oral digestion. After gastric digestion, the addition of 0.05% FA significantly increase the CEL content in the bioaccessible fraction and the addition of both 0.05% FA and 0.05% EC resulted in a significant increase in the CML content of the bioaccessible part. When the samples were subjected to intestinal digestion, both FA (0.2% and 0.5%) and EC (0.2% and 0.5%) significantly reduced the CEL content in the bioaccessible fraction, and 0.05% FA significantly increased the CML content in the bioaccessible fraction. Samples with 0.05% FA showed more pronounced differential effects on CEL and CML concentrations after intestinal digestion. In a simulated gastrointestinal digestion model, Feng et al. reported that the addition of fruit vinegar and bio-phenols inhibited the release of protein-AGEs. This inhibitory effect could be attributed to polyphenols alter the activity of digestive enzymes, thereby influencing the release of CML from glycoproteins, or to polyphenols forming complexes with CEL thereby affect their content [18]. The multifaceted influence of polyphenols on AGEs in the gastrointestinal tract needs to be considered from multiple aspects, such as polyphenol activity at different stages, the differential effects of various polyphenols, and the reactivity of polyphenols with AGEs and their precursors.

### 3.2. Variation of the α-DCs Content During the In Vitro Digestion Process

**Figure 3:**
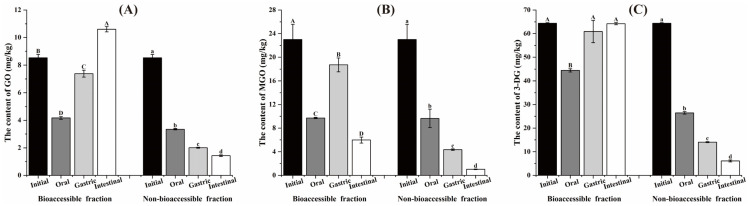
The content of GO (**A**), MGO (**B**) and 3-DG (**C**) in the bioaccessible and non-bioaccessible fractions of the sample after oral, gastric and intestinal digestion. Different capital letters and lowercase letters were used to indicate significant differences in the contaminant content of the bioaccessible and the non-bioaccessible fractions after different levels of digestion. The error bars were expressed in SD.

The reactive α-DCs, including 3-DG, GO, and MGO serve as key intermediates in the production of AA, 5-HMF, and AGEs. Monitoring changes in α-DCs levels can provide important information for understanding the formation patterns of these processing contaminants. Figure 3 showed the content of GO, MGO and 3-DG in the bioaccessible and non-bioaccessible fractions of the sample after oral, gastric and intestinal digestion without polyphenols. The initial contents of GO, MGO and 3-DG in biscuits were 8.5 mg/kg, 23.0 mg/kg and 64.4 mg/kg, respectively, aligning with the range reported for commercial biscuits: to be 4.8–26.0 mg/kg GO, 3.7–81.4 mg/kg MGO, and 8.5–385 mg/kg 3-DG [19,20]. Digestion significantly reduced α-DCs content in non-bioaccessible fractions. In bioaccessible fraction, GO and 3-DG showed an increasing trend across digestive stages, while MGO content increased after gastric digestion but decreased significantly after intestinal digestion. Hamzalıoğlu and Gökmen observed sample-dependent MGO changes after gastric digestion, followed by significant decreases after intestinal digestion [10]. Previous studies reported significant reductions in GO, MGO, and 3-DG concentrations after digestion, which is different from our results [21]. This discrepancy probably because our samples contained more precursors of 3-DG.

**Figure 4:**
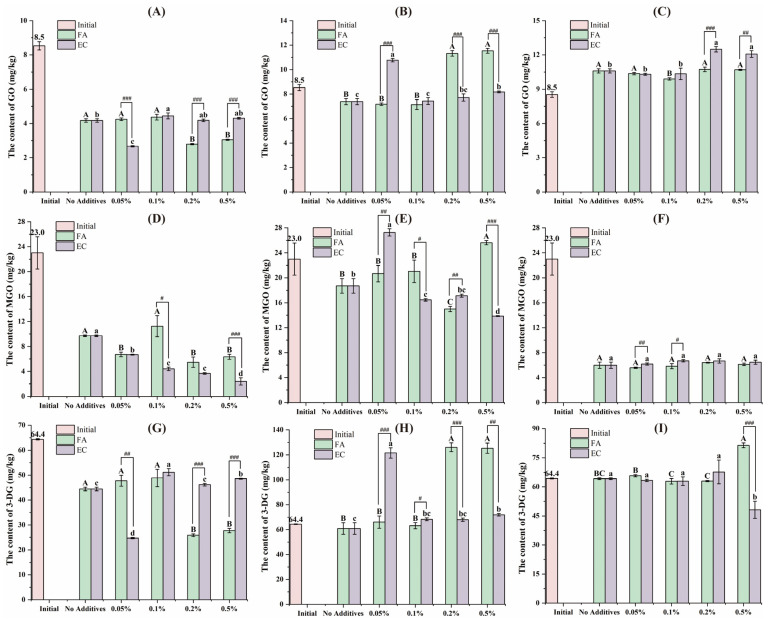
The GO content of the bioaccessible fraction of the samples after oral (**A**), gastric (**B**) and intestinal (**C**) digestion, the MGO content of the bioaccessible fraction of the samples after oral (**D**), gastric (**E**) and intestinal (**F**) digestion, and the 3-DG content of the bioaccessible fraction of the samples after oral (**G**), gastric (**H**) and intestinal (**I**) digestion. Different capital letters and lowercase letters were used to indicate significant differences between different concentrations of FA and EC. The # indicates a significant difference between FA and EC at the same level of digestion and concentration (#: *p* < 0.05, ##: *p* < 0.01, ###: *p* < 0.001). The error bars were expressed in SD.

Figure 4 showed modulatory effects of FA and EC on the levels of GO, MGO and 3-DG in the bioaccessible fraction across oral, gastric and intestinal digestion. After oral digestion, 0.2% and 0.5% FA and 0.05% EC significantly decreased the content of GO and 3-DG in the bioaccessible fraction; 0.05%, 0.2% and 0.5% FA significantly decreased the content of MGO in the bioaccessible fraction, and the higher the EC concentration, the lower the MGO content. After gastric digestion, 0.2% and 0.5% FA and 0.05% EC significantly increased the content of GO and 3-DG in the bioaccessible fraction. FA at 0.2% significantly decreased the content of MGO, and 0.5% FA significantly increased the content of MGO; the higher the EC concentration, the lower the content of MGO. After intestinal digestion, 0.1% FA significantly decreased the GO content in the bioaccessible fraction, while 0.2% and 0.5% EC significantly increased the GO content in the bioaccessible fraction. There was no significant effect of FA and EC on MGO content. EC at 0.5% significantly decreased 3-DG content in the bioaccessible fraction, while 0.5% FA had the opposite effect. At the stage of oral and gastric digestion, different polyphenols showed significant differences at the same concentration; however, after intestinal digestion, these differences became smaller. Polyphenols possess strong free radical-scavenging capacity and can inhibit lipid oxidation, thereby reducing α-DCs formation. This mechanism may underlie the inhibitory effects of polyphenols on the formation of α-DCs in the gastrointestinal tract [22].

### 3.3. Bioaccessibility of Contaminants After Digestion In Vitro

Table 1 shows the bioaccessibility of AGEs and α-DCs in the soluble fraction after intestinal digestion. A large amount of GO was generated during digestion, and its concentration in the bioaccessible fraction of the digested biscuits exceeded that in the initial biscuits, regardless of the addition of polyphenols. The bioaccessibility of GO in the digested biscuits without polyphenols was 124.3%. The effect of FA on GO bioaccessibility in the digested biscuit samples was not significant. However, GO bioaccessibility in the digested biscuit samples reached 146.4% and 141.5% after the addition of 0.2% and 0.5% EC, respectively. The significantly higher GO concentration in the digested biscuits compared to the initial biscuits could be attributed to the formation of GO by the *Maillard* reaction, glucose autoxidation, carbohydrate pyrolysis and lipid peroxidation during digestion [23].

The amount of MGO in the bioaccessible fraction of the digested biscuits was much lower than in the original biscuits, regardless of whether polyphenols were added or not. More than 80% MGO was present in the bioaccessible fraction of the digested biscuits, similar to the distribution of GO between the bioaccessible and non-bioaccessible fractions of the biscuit digest. The addition of FA or EC did not significantly affect the bioaccessibility of MGO in the digested biscuits. MGO can be produced by the *Maillard* reaction (reaction of glucose and fructose with amino acids), hexose autoxidation and oxidation of unsaturated fatty acids in lipids; however, MGO is also the main precursor for the production of AGEs, AA, which are consumed during digestion [24]. If MGO is consumed at a higher rate than produced, it can explain the decrease in MGO content during digestion.

The bioaccessibility of 3-DG after biscuit digestion was 99.7% when no polyphenols were added. In samples with FA addition, 0.5% FA increased the bioaccessibility of 3-DG in the soluble fraction to 126.6% and other concentrations had no significant effect, whereas in samples with added EC, 0.5% EC decreased the bioaccessibility of 3-DG in the soluble fraction to 74.8%, while other concentrations had no significant effect. FA and EC at 0.5% concentration showed different effects related to the type of polyphenols. Hamzalıoğlu and Gökmen [10] pointed out that α-DCs present in food can react with free amines and sulfhydryl groups of amino acids and proteins during simulated gastrointestinal digestion and confirmed the amino acids adducts of MGO and 3-DG like lysine-MGO. Polyphenols are highly reactive and may affect the formation of adducts of α-DCs with different amino acids during digestion, leading to differential changes in α-DCs during digestion.

Without the addition of polyphenols, the bioaccessibility of CEL and CML after biscuit digestion was 89.5% and 103.4%, respectively. With the addition of polyphenols, it was observed that 0.05% of both FA and EC significantly increased the bioaccessibility of CEL and CML. This phenomenon may have arisen because polyphenols facilitated the conversion of some precursors in the food matrix into AGEs or promoted the release of bound AGEs [25], or they may have inhibited the binding of CEL and CML to other substances.

### 3.4. The Content of Contaminants in the Insoluble Fraction After Digestion In Vitro

Table 2 shows the content of AGEs and α-DCs in insoluble fractions after in vitro digestion. Most of the contaminants were transferred and dissolved in the bioaccessible fractions during digestion, and only a small amount of contaminants were still attached to the non-bioaccessible fraction. Among them, the contents of GO in the insoluble component after digestion accounted for about 10% of the total content after digestion, while the contents of MGO, CEL and CML accounted for less than 20%, and the content of 3-DG only accounted for less than 10%. Although the proportion of contaminants in the non-bioaccessible fraction is small, its existence can still pose a risk to human health. 

Digestion within the small intestine (duodenum) does not conclude digestive process; undigested food residues transit to the large intestine (colon) for further fermentation mediated by intestinal microbiota. The rate of intestinal peristalsis, the activity of the intestinal microbiota, and the composition of undigested food residues may collectively affect colonic digestion. Colonic fermentation can lead to the release of certain substances, such as bound polyphenols, thereby increasing their bioaccessibility [26]. Interactions between polyphenols and contaminants may also continue during colonic fermentation. Data from Li et al. showed that fiber-bound polyphenols were able to scavenge 35.8% to 45.2% of MGO and 19.3% to 25.4% of GO during fermentation and play a key role in scavenging carbonyl compounds [27]. In the future, the transformation of contaminants during intestinal fermentation can be studied in detail to better assess their potential impact on human health.

### 3.5. Structural Identification of Adducts Formed During Digestion In Vitro

As two naturally occurring polyphenolic antioxidants, FA and EC have been proven to affect the formation of multiple contaminants in *Maillard* reaction and to form mono-, di-or multi-adducts with various contaminants and amino acids. Researchers identified epicatechin-mono-MGO, epicatechin-mono-GO and di-epicatechin-mono-acetaldehyde in ultra-high temperature stored milk [28]. In addition, some researchers identified cysteine-MGO adduct and cysteine-GO adduct, and demonstrated that these adducts exhibit much lower cytotoxicity [29]. To elucidate the mechanism underlying polyphenols interactions with thermal processing contaminants in the gastrointestinal tract, we conducted a preliminary structural characterization of the digestion products using UPLC-Q-TOF-MS. Further confirmation of the structure needs to be achieved through nuclear magnetic resonance and other methods.

**Figure 5:**
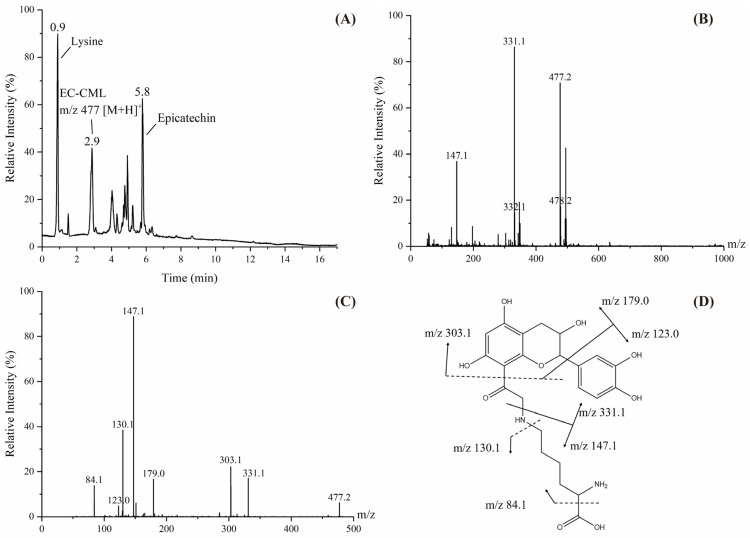
Total ion chromatogram (**A**), primary mass spectrum (**B**), secondary mass spectrum (**C**) and fragmentation pathway (**D**) of the adduct with *m*/*z* of 477. The figure only suggests substitution on C8, although substitution may also occur on C6.

In the simulated reaction system of GO-Lys-EC, the molecular ion peak of the EC adduct with *m*/*z* 477 was detected (Figure 5). The fragment ions of the adduct were *m*/*z* 331.1, 303.1, 179.0, 147.1, 130.1, 123.0, 84.1 based on the secondary mass spectrum. From this, it can be deduced that the adduct was formed when one molecule of CML undergoes a substitution reaction at the C6 or C8 positions of the A ring of EC and then removes one molecule of H_2_O [290 + 204 − 18]. EC belongs to the flavonoid polyphenols, and the C6 and C8 positions of the A ring of flavonoids are the active sites of the reaction, so the adduct may have two substitution sites. The presence of this adduct demonstrated that α-DCs can react with lysine to form AGEs and highlighted the interaction between polyphenols with AGEs.

**Figure 6:**
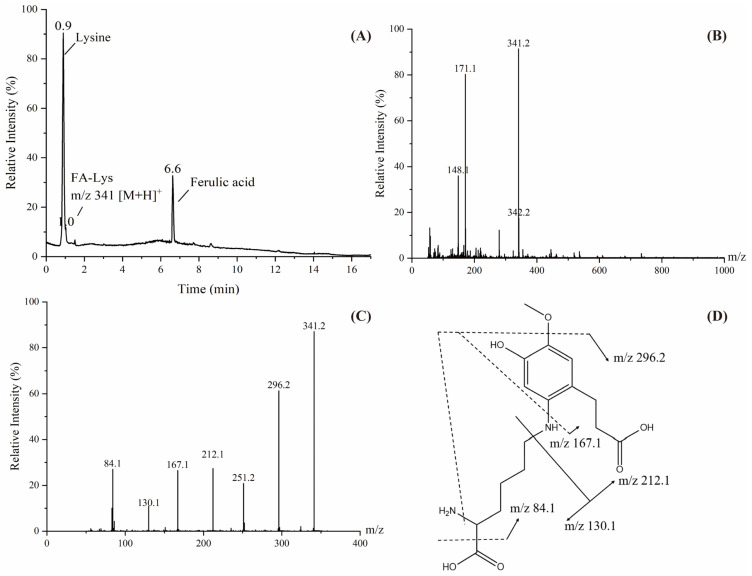
Total ion chromatogram (**A**), primary mass spectrum (**B**), secondary mass spectrum (**C**) and fragmentation pathway (**D**) of the adduct with *m*/*z* of 341.

In the simulated reaction system of MGO-Lys-FA, in addition to the molecular ion peaks of Lys and FA at 0.9 min and 6.63 min, respectively, the molecular ion peak with *m*/*z* of 341 was found at 1.03 min (Figure 6), indicating that the molecular weight of the adduct was 340. Therefore, it can be inferred that the adduct might be formed by the addition of one molecule of FA with one molecule of lysine and the elimination of one molecule of H_2_ to complete the double bond reduction of FA to form [194 + 146 − 2 + 2]. The fragment ions were *m*/*z* 296.2, 212.1, 167.1, 130.1, 84.1, etc., where 130.1 and 84.1 were two characteristic fragment ion peaks of lysine, 167.1 was the FA part of the adduct removing a molecule of methoxy after reducing, and 212.1 was the part of the adduct formed by the break of the FA-linked lysine amino group, with the adduct removing the methoxy and amino to produce the ion of *m*/*z* 296.

## 4. Conclusions

This study employed the standardized INFOGEST in vitro digestion protocol to systematically characterize the bioaccessibility of AGEs (CML, CEL) and α-DCs (GO, MGO, 3-DG) in biscuits and evaluated the modulatory effects of FA and EC as well as to explore the interaction mechanism between polyphenols, amino acids, and contaminants in the digestive tract through structural analysis of adducts in the products. It was found that most of the AGEs and α-DCs were transferred to the bioaccessible fraction after intestinal digestion, with less than 20% retained in the non-bioaccessible fraction. In bioaccessible samples, FA at 0.05% (*w*/*w*) significantly increased the CEL and CML contents after intestinal-digestion. FA at 0.2% and 0.5% and 0.05% EC significantly decreased the GO and 3-DG contents during oral digestion but significantly increased the contaminants bioaccessibility after gastric digestion. The EC concentration and the MGO content showed a negative correlation during oral and gastric digestion. After intestinal digestion, the effects of polyphenols type/concentration on α-DCs diminished, with most treatments increasing GO and 3-DG bioaccessibility, but reducing MGO levels independent of dose. In addition, LC-QTOF-MS analysis confirmed the formation of FA-lysine adducts and EC-CML adducts, demonstrating the polyphenols reactivity with amino acids and processing contaminants. Future research should focus on obtain pure adducts formed during gastrointestinal digestion, as well as to study the transformation of contaminants during colonic fermentation in order to better assess their potential impact on human health.

## Figures and Tables

**Table 1:** Bioaccessibility of AGEs and α-DCs in the soluble fraction after in vitro digestion.

		CEL (%)	CML (%)	GO (%)	MGO (%)	3-DG (%)
No Additives		89.5	103.4	124.3	26.0	99.7
FA	0.05%	102.1	132.5	121.4	24.2	102.1
	0.1%	84.5	102.3	116.0	25.3	97.7
	0.2%	81.4	99.7	125.8	27.8	97.9
	0.5%	81.5	103.0	125.4	26.6	126.6
EC	0.05%	89.7	107.7	120.7	26.9	98.4
	0.1%	87.7	105.7	121.2	29.1	97.8
	0.2%	83.5	103.5	146.4	29.0	105.2
	0.5%	82.1	97.6	141.5	28.1	74.8

**Table 2:** AGEs and α-DCs content of insoluble fraction after in vitro digestion.

		CEL (mg/kg)	CML (mg/kg)	GO (mg/kg)	MGO (mg/kg)	3-DG (mg/kg)
Initial		17.6	21.7	8.5	23.0	64.4
No Additives		3.4	4.3	1.4	1.0	6.1
FA	0.05%	3.0	3.9	1.5	1.1	5.8
	0.1%	3.0	4.3	1.4	1.1	5.1
	0.2%	3.5	4.6	1.6	1.1	6.1
	0.5%	3.0	4.2	1.4	1.2	4.9
EC	0.05%	3.0	3.7	1.4	1.1	6.0
	0.1%	2.9	3.5	1.3	0.9	6.2
	0.2%	3.5	4.1	1.7	1.2	3.3
	0.5%	3.4	4.2	1.3	1.0	1.2

## Data Availability

The original contributions presented in the study are included in the article/Supplementary Materials, further inquiries can be directed to the corresponding author.

## Supplementary Materials

The following supporting information can be downloaded at: https://www.mdpi.com/article/10.3390/foods14081429/s1, Figure S1: The calibration curves of CEL (A), CML (B), GO (C), MGO (D) and 3-DG (E). The abscissa represented the ratio of the concentration of the target substance to that of the internal standard, while the ordinate represented the ratio of the peak area of the target substance to that of the internal standard; Figure S2: The representative chromatograms of AGEs in bioaccessible and non-bioaccessible fractions after digestion in the oral, stomach and intestine; Figure S3: The representative chromatograms of α-DCs in bioaccessible and non-bioaccessible fractions after digestion in the oral, stomach and intestine.
