# Supplementary material for: In Vitro Digestion Patterns of Advanced Glycation End Products and α-Dicarbonyls in Biscuits and the Modulatory Effects of Ferulic Acid and Epicatechin
Source: Foods. 2025 Apr 21;14(8):1429. doi: 10.3390/foods14081429 (PMC12027215; doi:10.3390/foods14081429)

Supplementary File

Figure S1. The calibration curves of CEL (A), CML(B), GO(C), MGO (D) and 3-DG (E). The abscissa represented the ratio of the concentration of the target substance to that of the internal standard, while the ordinate represented the ratio of the peak area of the target substance to that of the internal standard.

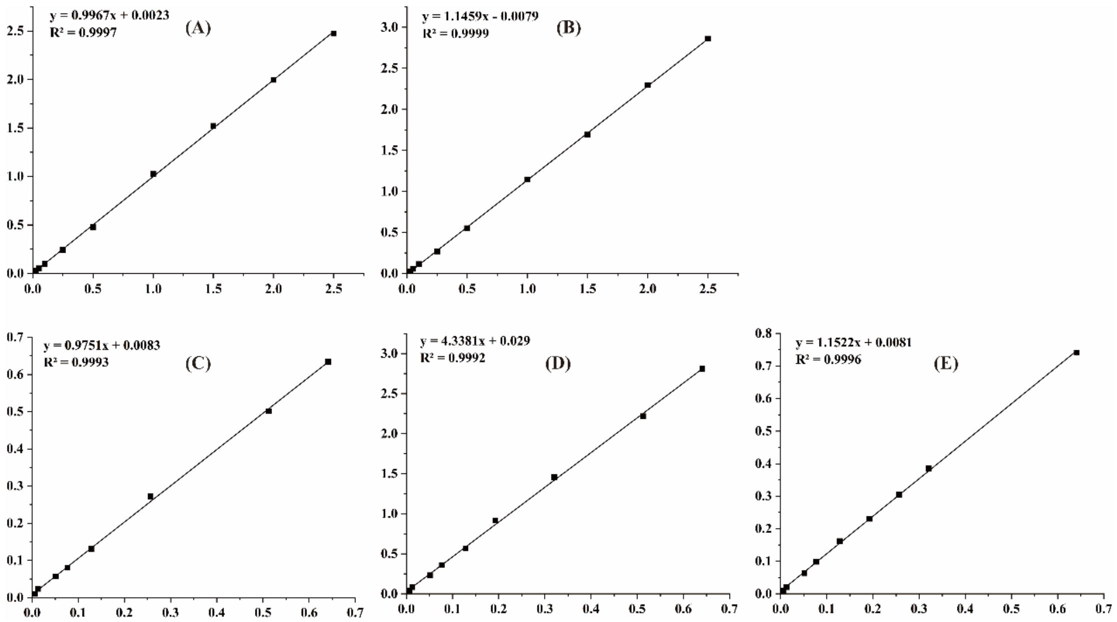

Figure S2. The representative chromatograms of AGEs in bioaccessible and non-bioaccessible fractions after digestion in the oral, stomach and intestine.

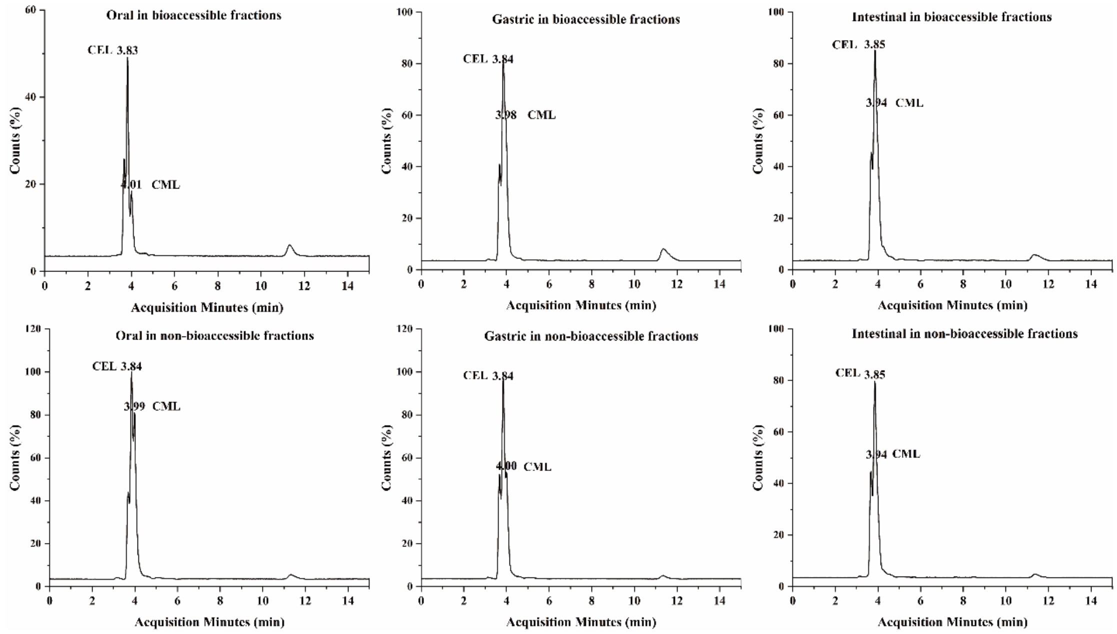

Figure S3. The representative chromatograms of α-DCs in bioaccessible and non-bioaccessible

fractions after digestion in the oral, stomach and intestine.

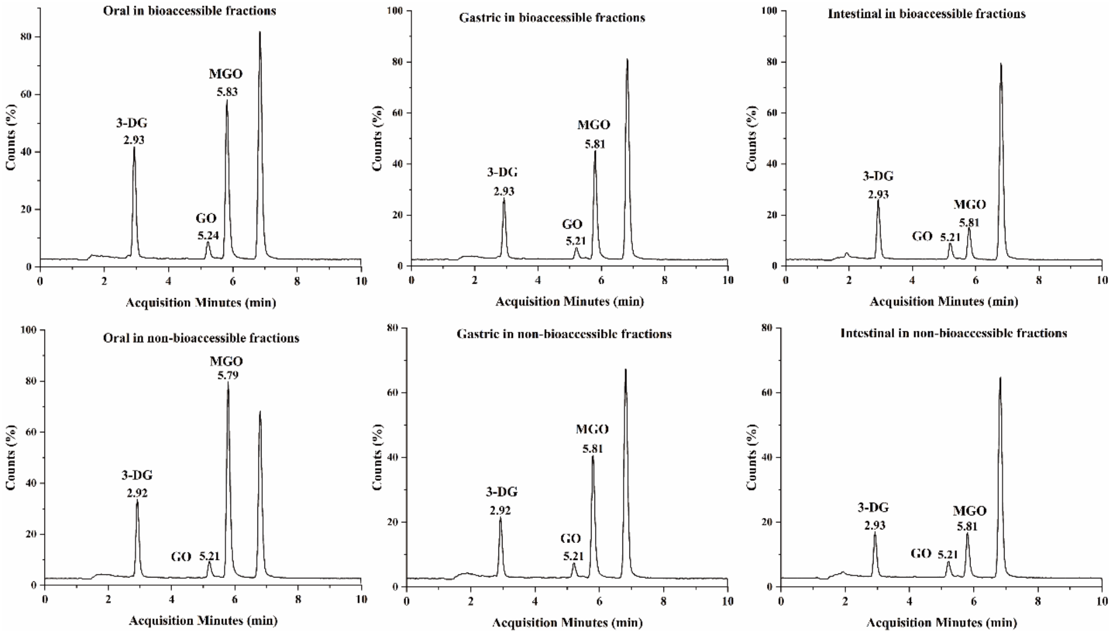

Supplement: Supplementary file 1 [file foods-14-01429-s001.zip › foods-3530573-supplementary.pdf]
